# Supplementary material for: From Inference to Ritual: Why Frequentist Statistics Became Hard to Understand and Teach in Medicine
Source: J Eval Clin Pract. 2026 Sep 19;32(6):e70608. doi: 10.1111/jep.70608 (PMC13589657; doi:10.1111/jep.70608)
Supplement: Supplementary file 1 [file JEP-32-0-s001.pdf]

Medical Education

Mythology

From Inference to Ritual: Why Frequentist Statistics Became Hard to Understand and Teach in Medicine

Submission ID 8a24cafb-07e5-4713-bf82-b938c00ae437

Manuscript ID 2931300

Submission Version Initial Submission

PDF Generation 13 Apr 2026 02:39:30 EST by Atypon ReX

Files for peer review

All files submitted by the author for peer review are listed below. Files that could not be converted to PDF are indicated; reviewers are able to access them online.

Name	Type of File	Size	Page
Manuscript_Lega_Chevret_ME.docx	Anonymized Main Document - MS Word	61.2 KB	Page 3
Figure1.pdf	Figure	139.1 KB	Page 22
Figure2.pdf	Figure	40.5 KB	Page 23

From Inference to Ritual: Why Frequentist Statistics Became Hard to Understand and Teach in Medicine

ABSTRACT

Introduction Frequentist statistical tools still structure a large part of biomedical research, regulatory evaluation, and medical teaching, even though their correct interpretation remains uncertain among students, clinicians, and readers of the literature. This misunderstanding is usually attributed to pedagogical shortcomings or individual cognitive errors. This article examines the structural reasons for this persistent misunderstanding. It argues that it arises from a combination of historical, methodological, semantic, and institutional factors rather than from a direct lack of technical training.

Methods The paper uses a selective narrative and historical analysis of the Bayes–Fisher–Neyman trajectory, of the language used to describe major frequentist tools, of the critiques of their ritualized use, and of their institutionalization in editorial, regulatory, and curricular settings. Its purpose is synthetic and argumentative, with a focus on medical education.

Main findings The article argues that, in Fisher's framework, the p-value was not an isolated signal but one element within a dense experimental architecture based on study design, sufficient statistics, and exhaustive extraction of information. The later shift toward a procedural and decision-oriented meaning strengthened its large use as a simple decision tool but made its frequentist meaning less natural. This opacity is further reinforced by ambiguous terminology, by the separation between statistics and methodology in teaching, and by ritualization through editors, agencies, and curricula.

Conclusion Misunderstanding of frequentism in biomedicine should be seen as a structural problem of intelligibility. A realistic educational response requires reconnecting calculation, study design, history, epistemology, and language, so that teaching moves away from ritual use of tools and toward a more robust understanding of their meaning and limits.

Keywords: Frequentism, P value, Inference, Medical education, Epistemology, Randomization

INTRODUCTION

The paradox is familiar. Frequentist tools remain dominant in biomedical publications, regulation, and teaching, yet their correct interpretation remains highly heterogeneous [1]. The p-value is still often misdescribed as the probability that the observed data occurred "by chance," or even that the null hypothesis is true. In 2016, the American Statistical Association clarified that a p-value is neither the probability that the null hypothesis is true nor a direct measure of effect importance [2]. More recently, the International Committee of Medical Journal Editors recommended reporting findings with measures of uncertainty, such as confidence intervals, rather than relying only on hypothesis tests [3]. Despite these recommendations, p-values remain routine in biomedical literature. Ioannidis showed that among PubMed articles reporting p-values, 96% of full texts reported values ≤ 0.05, whereas only 4% reported confidence intervals for all presented effect sizes [4]. The issue affects the core of evidential practice.

We hypothesized that this opacity is not accidental. It likely arises from the combined effects of erased conceptual history, teaching centered on thresholds rather than spaces of reasoning, invisibility of the knowledge embedded in study design, misleading terminology, and the later institutionalization of routines that converted a logic of inference into a social technology for sorting results. Modern frequentism is thus often taught in a thinner form than the historical conditions that originally gave it meaning.

HISTORICAL REMINDER: BAYES, THEN FISHER, IN A WORLD OF SCARCE DATA AND STRONG METHODOLOGICAL DEMANDS

The contrast between Bayes and Fisher should not be reduced to a simple opposition between subjectivity and objectivity. In a Bayesian framework, prior knowledge is explicit. The probability of a hypothesis H given the data D, written as P(H|D), depends not only on the probability of the data given the hypothesis P(D|H), named the 'likelihood', but also on the probability assigned to the hypothesis before the data are observed, P(H), named the 'prior" [5]. In the experimental frequentist world, apart the likelihood, the prior knowledge does not disappear; it changes location. Fisher places it not in a formal prior distribution but in the experimental control, model specification, sufficient statistics, and disciplined study design [6]. The key point of his 1936 address is straightforward: one must learn to apply reason to observational facts that are few in number and imperfect in precision, while drawing from them

only those inferences that the observations warrant [7]. The central issue is therefore not the mechanical production of a threshold, but the rigorous limitation of inference.

Within this framework, Fisher's p-value is not an autonomous object. It belongs to a system in which the quality of inference depends on whether all relevant information has been properly used. Fisher emphasizes the importance of sufficient statistics and argues that an inferential argument is acceptable only if the relevant information has been exhaustively mobilized; otherwise, "*anything can be proved by statistics*" [7]. This is not a rhetorical detail. It means that a p-value has meaning only within a strongly constrained methodological environment, one in which the design protects the data and the analysis is not expected to repair a weak study after the fact. Armitage recalls that Fisher treated randomization as a key ingredient in the validity of statistical tests, and that Hill later carried this discipline into comparative clinical trials to reduce systematic bias between groups [6].

Precision is therefore essential. The p-value is not a posterior probability that the null hypothesis is true or that the alternative hypothesis is true. The null hypothesis, usually written as H0, refers to the hypothesis of no effect, no association, or no difference. The alternative hypothesis, usually written as H1, refers to the competing possibility that an effect, association, or difference exists. Fisher's point is not to assign direct probabilities to these hypotheses after seeing the data. Rather, in a highly structured experimental world, the p-value participates in a logic of interpreting the observed case, and this logic is closer to the practical needs of a researcher than the later purely procedural meaning of statistical inference [8]. It is this functional proximity, not any formal equivalence with Bayes, that matters here [7,9,10].

WHY FREQUENTISM BECAME COGNITIVELY UNNATURAL

The difficulty of modern frequentism lies first in the way it is taught. Students often learn the concept of the tail area under the null hypothesis (Figure 1) and the significance threshold. Sometimes, they also learn about statistical power. However, these concepts are taught without a clear representation of the space formed by the null and alternative hypotheses, without a clear picture of true and false positives and negatives, and without a visible role for prior plausibility. Put differently, students learn symbols before they learn the geometry of inference. Gigerenzer showed that the "null ritual" does not properly belong to either Fisher or Neyman–Pearson, but rather to a hybrid pedagogical and practical routine that removes judgment precisely where statistical theories require it [11]. This loss of judgment is reinforced by the fact that teaching

rarely compares what a Fisher significance test offers, what a Neyman–Pearson procedure requires, and what a Bayesian reading adds [12].

As a result, users spontaneously reconstruct what is missing. In 1987, Browner and Newman provided an especially helpful diagnostic analogy for physicians [13]. In their framework, α is the pre-specified probability of a type I error, that is, a false positive; $1 - \beta$ is power, the probability of detecting an effect when one truly exists; β is the probability of a false negative [13]. Prior plausibility plays a role analogous to prevalence in diagnosis. It shows why readers want to locate a result in a space where false positives, false negatives, and prior plausibility coexist [14].

However, a further limit must be stated explicitly. Much of the empirical literature on misinterpretation originates from psychology and the social sciences. Transferring these findings to medical education requires caution [15]. This does not invalidate the argument, because there is medicine-specific evidence. In a previous multicenter survey of internal medicine residents, the mean score for understanding biostatistics and interpretating results was only 41%. Additionally, 75% of respondents stated that they did not understand all the statistics they encountered in journal articles [16]. While findings from other disciplines may therefore illuminate general cognitive mechanisms, their generalization to medical training should be supported by studies conducted with health professionals or medical learners, whenever possible.

AN AMPUTATED PEDAGOGY: MUCH CALCULATION, LITTLE METHODOLOGY, NO EPISTEMOLOGY

The problem lies not only in the logic of the tools, but in the segmentation of teaching. In many biomedical curricula, statistics are taught as a technical skill, whereas methodology, critical appraisal, history of experimentation, and epistemology remain scattered or absent. Yet the knowledge that structures frequentist interpretation is already present upstream: choice of comparator, eligibility criteria, hierarchy of hypotheses, important effect sizes, sources of heterogeneity, and the balance between α and β. Kubiak and Kawalec argued that, in the frequentist tradition, prior information is redistributed into study design and calibration commitments rather than eliminated [3].

Standard training is therefore incomplete. It provides conditional numerical outputs without teaching students to read the protocol as part of the inferential argument. This creates a paradox:

students are taught instruments said to produce evidence-based results, while the methodological commitments that make those outputs interpretable remain hidden. Fisher argued the reverse: inference becomes arbitrary when relevant information has not been considered [1]. What must be taught is not only the calculation, but also the place where structured prior knowledge resides within a frequentist inquiry.

WORDS AGAINST COGNITION: MISLEADING SEMANTICS AND NEUTRALIZED REASONING

The difficulty is also lexical. The term "*p-value*" does not indicate which probability is being discussed. "*Confidence interval*" almost irresistibly suggests confidence in the observed interval itself. "*Statistical significance*" suggests importance, although it usually refers only to a conditional measure of compatibility between the data and H0 under a model. This ambiguity is not trivial: in ordinary language, "*significant*" means important, whereas in statistics "*not significant*" means only that the data are insufficient to reject H0 [17]. The problem is therefore not mere using ignorance, but also a collision between ordinary and technical language [18].

The most useful response is probably not to invent an entirely new jargon, but rather to introduce a parallel pedagogical vocabulary. Rafi and Greenland propose replacing "*confidence*" and "*significance*" with "*compatibility*" and "*surprise*", precisely because the problem is cognitive before it is mathematical [18]. This aligns with the ASA statement that the p-value concerns the data under H0 and an assumed model, not the probability of the hypothesis itself. In practice, one could therefore teach the p-value as a tail-area measure of compatibility, and a confidence interval as a region of high compatibility with the data and background assumptions, without pretending that the institutional vocabulary can be abolished immediately [18].

FROM INFERENCE TO RITUAL: REGULATORY, EDITORIAL, AND CURRICULAR INSTITUTIONALIZATION

The opacity of frequentism persisted because it became institutionalized. Bradford Hill, a central figure in epidemiology and randomized trials, warned in 1965 against the ritualization of significance testing in editorial practice. He noted that some editors returned articles because significance tests had not been applied, and that "*the glitter of the t table*" could distract from weaknesses in the data [19]. The critique of ritualization is therefore old. It was formulated by a central architect of modern methodology [19].

This ritualization was later reinforced by textbooks and academic culture. Gigerenzer's "null ritual" is not just a logical mistake; it is a socially stabilized practice. This ritual is accompanied by "*delusions*," including the replication delusion, which states that one minus the p-value would represents the probability that a result will replicate. In the studies he reviewed, 20% of psychology statistics instructors, 39% of non-specialist instructors, and 66% of students held this belief [10]. Gigerenzer and Marewski later described "*surrogate science*," in which the search for statistically significant p-values replaces scientific judgment and encourages borderline misconduct [20]. Greenland, for his part, described three cognitive distortions: dichotomania, nullism, and statistical reification [18].

The institutional dimension is equally clear. ICH E9 explicitly defines "frequentist methods" as methods such as significance tests and confidence intervals. It also recommends reporting exact p-values rather than referring only to critical values [21]. The ICMJE asks authors to report results with appropriate measures of uncertainty and to avoid exclusive reliance on hypothesis tests [3]. Such formalization improved reporting discipline, but it also helped transform statistical concepts into administrative obligations. Temple, describing decision-making at the Food and Drug Administration, showed that the real decisions are never based on p-values alone; they already involve coherence, plausibility, and supporting confirmation [22]. The routine use of p-values was therefore not only taught, but also administratively consolidated.

CONSEQUENCES AND CONCRETE PEDAGOGICAL PROPOSALS

The consequences of this misunderstanding are substantial. First, it promotes a binary reading of evidence, replacing analysis of effect size, precision, plausibility, and limitations of validity, with crossing a threshold. Second, it encourages spontaneous quasi-Bayesian interpretations, but without an explicit prior or a coherent inferential architecture. Finally, it makes it more difficult to understand what gives a study its strength.

One initial proposal is to reconnect the teaching of inferential statistics with the evidential strength that justifies conducting a study in the first place. This requires an explicit representation of the two probability spaces shown in Figure 1. The Bayesian space, shown in panels A1 and A2, makes the dependence of the result on prior plausibility visible. The frequentist space, shown in panel B, displays the sampling distributions under H0 and H1, as well as the α region, the β region, and the p-value (Figure 1). Juxtaposing the two spaces makes explicit what standard teaching usually leaves implicit: the inferential value of a significant

result depends on more than crossing a threshold; it also depends on the full set of conditions that make that crossing meaningful. Pedagogically, this also enables the teaching of the Neyman–Pearson framework through the Browner and Newman analogy [13]. When P(H1) (i.e. prior plausibility in Bayesian perspective) is 0.50, α is set at 0.05, and β at 0.20, so that power is 0.80, the positive predictive value of a significant result, that is, the probability that H1 is true given a significant finding, is 0.400 divided by (0.400 + 0.025), or approximately 0.941. This corresponds to a false positive rate of approximately 0.059, close to the conventional 0.05 threshold (Figure 2) [13]. But this convergence requires three demanding conditions: absence of major systematic bias, effective control of α and β as specified in the protocol, and a genuinely strong prior plausibility near 0.50 grounded in robust preliminary evidence. These conditions are rarely met simultaneously, and teaching seldom makes them explicit.

A second proposal is to connect each statistical result to its methodological context, including evidence supporting the hypothesis study, inclusion criteria, core study design, comparator, estimand, bias, multiplicity, plausibility, and subgroup structure. The main support for this proposal comes from the literature showing that, in frequentist reasoning, the inferential force of a result depends on design-embedded commitments rather than on the numerical output alone.

A third proposal is to introduce a brief but explicit module on the history of Bayes, Laplace, Fisher [7], Neyman [23], and Hill [19]. This module would not be for antiquarian purposes, but rather to restore the meaning of the main conceptual bifurcations (Table 2). The main rationale behind this proposal is epistemological and is based on the hypothesis that persistent misinterpretations are more likely to occur when learners are unaware of the historical divergence between evidential, procedural, and decision-oriented frameworks [24]. This causal relationship has not been tested empirically and should be considered as a hypothesis rather than an established result.

A fourth proposal is to make the semantic pitfalls of statistical language explicit in teaching. Terms such as "*confidence interval*," "*statistical significance*," and "*p-value*" should be presented not as transparent labels. Rather, they should be presented as expressions whose ordinary-language meaning can mislead interpretation unless their technical meaning is carefully unpacked (Table 1).

These proposals should be positioned in relation to the existing educational literature. In statistics education, curricula centered on statistical thinking and simulation, associated especially with Cobb [25], have shifted the focus of learning from formula manipulation to the broader logic of inference. The present proposals add historical, semantic, and institutional layers to that cognitive reorientation

CONCLUSION

Frequentism is difficult to understand not because it is overly mathematical, but because it is often taught outside of its experimental, historical, and semantic context. Laplace, Fisher, and Neyman did not approach the same inferential problem in the same way. Subsequent editorial and regulatory standardization transformed the logic of inference into a more ritualized practice.

Under these conditions, common misunderstandings of p-values and confidence intervals are not merely individual errors. Rather, they are largely produced and perpetuated by the manner in which these tools are taught and used. Explaining the historical divergence between evidential, procedural, and decision-oriented frameworks will not resolve all difficulties of understanding. Nevertheless, the main conclusion stands: a more intelligible teaching of frequentism should reconnect statistical tools to study design, to the history and logic of biomedical evidence, and to the methodological judgments that procedures alone cannot replace.

Declaration of competing interests: the authors have no competing interests to declare

Ethics approval: Ethical approval was not required for this work because it is a commentary based exclusively on published literature and involves no human participants, patient data, or animal subjects.

Funding statement: none

Data statement: no new data were generated or analyzed for this commentary

Declaration of generative AI and AI-assisted technologies: During the preparation of this work, the first author used ChatGPT (OpenAI 5.4) to assist with language editing and phrasing

TABLES

Table 1. Canonical statistical terms, their main semantic teaching traps, and possible pedagogical alternatives using analogy with diagnostic test. The plain-language explanations in the third column deliberately tolerate a degree of simplification where this aids comprehension without introducing major conceptual errors; they are not intended to replace standard statistical terminology in regulatory or editorial practice.

Canonical name	Semantic trap to teach explicitly	Pedagogical alternative with teaching note
P-value	Often misread as the probability that the null hypothesis is true, or as the probability that the result will replicate	*Conditional probability; how surprising are these data if there were really no effect? It is a tool for assessing evidence against the null of no effect*
Statistical significance	Suggests substantive importance, truth, or decisiveness, although it only it is determined by comparing the p-value to a pre-specified compatibility threshold	*Threshold-based incompatibility of data with no effect; the result is unusual enough to cross the statistical cutoff we chose in advance*
Non-significant result	Often misread as evidence of no effect, rather than as insufficient incompatibility with the null model	*Statistically inconclusive result; the study did not give strong enough evidence to clearly move away from no effect*
Confidence interval	Suggests a level of confidence in the observed interval itself, rather than a procedure with repeated-sampling properties	*Compatibility interval; this is the range of effect sizes that fit the data reasonably well*
95% confidence level	Often misread as meaning a 95% probability that the true value lies in the observed interval	*Procedure with 95% long-run coverage; if we repeated this kind of study many times, about 95 out of 100 intervals made this way would contain the true value*
Null hypothesis	Sounds like an empty, implausible, or 'false by default' hypothesis rather than a formal reference model	*Reference hypothesis; a formal 'no effect' baseline used to calculate how surprising the data are under this hypothesis. Unlike a Bayesian approach, frequentist tools do not tell you how likely this baseline is to be true — they only tell you how compatible your data are with it*

Canonical name	Semantic trap to teach explicitly	Pedagogical alternative with teaching note
Alternative hypothesis	Often taught as the automatically preferred or 'desired' claim, without clarifying its role in design, power, and interpretation	*Competing effect hypothesis justifying the study; this is the competing idea that a real effect may exist*
Power (1- Probablity of type II error)	Often treated as a technical planning quantity only, with indirect connection to interpretation (see diagnostic test analogy)	*Sensitivity; if a real effect exists, this is the probability of detecting it; the risk of missing it is 1 – power=beta*
Probability of Type I error (alpha)	Often treated as a technical planning quantity only, with indirect connection to interpretation (see diagnostic test analogy)	*1 − specificity; this is the false-alarm rate we accept before seeing the data*

Table 2. Key historical milestones in statistical inference and their pedagogical implications for biomedical teaching

**Date**	**Person**	**Key event**	**Pedagogical consequence**
1763	Thomas Bayes	Posthumous publication of Bayes's essay on inverse probability	Establishes the classic Bayesian question: how should prior plausibility be updated by data?<br>Pedagogically, this is the clearest starting point for showing that some inferential frameworks ask directly about the probability of a hypothesis given observed data
1814	Pierre-Simon Laplace	Generalises Bayesian inversion and develops a probabilistic theory of induction, causes, and evidence in *Essai philosophique sur les probabilités*	Stabilises the Bayesian grammar of inference.<br>Useful for teaching that prior knowledge was originally explicit in the formalism rather than hidden in study design or background assumptions
1900	Karl Pearson	Describes the chi-square distribution and its use for goodness-of-fit and association testing	Makes probability-based significance assessment more operational in large samples<br>Useful for distinguishing early model-checking and discrepancy testing from later small-sample and design-based inferential traditions
1908	William S. Gosset ("Student")	Introduces the t test for small samples while working at Guinness	Begins the modern logic of valid inference from small samples in Guinness facility<br>Discussion with R. Fisher regarding experimental design, including randomization<br>Highly relevant for medicine, where decisions are often made under scarcity rather than abundance of data
1914	Karl Pearson	Publishes Tables for Statisticians and Biometricians and consolidates a table-based statistical culture	Helps standardise practical testing. Also sets the background for later tensions over access to tables and the portability of statistical methods for non-specialists
1922	Ronald A. Fisher	Publishes "On the Mathematical Foundations of Theoretical Statistics" and formalises likelihood-	Shifts the centre of gravity from probability of hypotheses to information in the sample.<br>Essential for teaching why Fisher's inferential framework is neither Bayesian

Date	Person	Key event	Pedagogical consequence
		based statistical reasoning, p-value	nor identical to later Neyman–Pearson testing
1925	Ronald A. Fisher	Publishes *Statistical Methods for Research Workers*, diffusing significance testing to experimental scientists and providing practical critical values, especially at 5% and 1%	Later historical accounts report that Fisher was not allowed to reproduce Pearson's full tables, which likely encouraged a compact critical-value approach. Pedagogically, this helps explain why 0.05 and 0.01 became portable thresholds in applied science
1926	Ronald A. Fisher	Explicitly states his preference for the 5% level as a convenient low standard of significance in *The arrangement of field experiments*	Important for teaching that Fisher's threshold language was pragmatic and conventional, not a universal law of nature
1933	Jerzy Neyman and Egon S. Pearson	Formalize hypothesis testing with H0, H1, type I error, type II error, and power	Changes the question from "what do these data suggest?" to "what rule controls errors in repeated use?" Central for teaching why modern frequentism is procedural and often counterintuitive for beginners
1935	Ronald A. Fisher	Publishes *The Design of Experiments* and formalizes randomization, blocking/stratification, and design-based analysis	Shows that inferential meaning depends on design, not only on computation. Pedagogically crucial for reconnecting p values and intervals to protocol architecture.
1937	Jerzy Neyman	Develops confidence interval theory in a long-run frequentist framework	Provides the basis of the modern confidence interval, but also one of its main pedagogical traps: the interval concerns procedure performance in repeated sampling, not directly the probability that the true value lies in the observed interval.
1948	Austin Bradford Hill and the MRC streptomycin investigators	Conduct the streptomycin trial, commonly regarded as the first modern randomized clinical trial	Brings inferential statistics into the architecture of clinical experimentation. Ideal for teaching that randomization is part of evidence construction, not a decorative statistical add-on.
1965	Austin Bradford Hill	Publishes "*The Environment and Disease: Association or Causation?*" and warns	Joins causal reasoning to methodological judgment and anticipates later critiques of p-value ritualization. Pedagogically valuable because it shows

Date	Person	Key event	Pedagogical consequence
		against routine editorial demands for significance tests	that worries about mechanical testing are longstanding within biomedicine itself.

FIGURES

Figure 1. Prior plausibility and sampling distributions as two complementary representations of the inferential space. Panels A1 and A2 show a weighted H0/H1 space in which the proportions of true positives, false positives, true negatives, and false negatives vary according to prior plausibility; they make visible the question readers often ask implicitly, namely how likely a significant result is to correspond to a true effect. Panel B shows the standard frequentist sampling space under H0 and H1, with α, β, power, and the p-value, thereby illustrating that the formal output of frequentist testing is not identical to the broader inferential question that users often try to answer.

Figure 2. Figure 2. False positive risk, prior plausibility, and the diagnostic test analogy. Panel A shows how false positive risk (FPR) varies with the prior plausibility of H1 for fixed α and different power values. Panel B summarizes the correspondence between diagnostic testing and randomized trials: prevalence maps to prior plausibility, sensitivity (Se) to power, 1−specificity (Spe) to α, and positive predictive value to 1−FPR. Panel C gives a concrete worked example showing how these quantities are read directly from the figure for one selected scenario

REFERENCES

1 Gelman A, Stern H. The Difference Between "Significant" and "Not Significant" is not Itself Statistically Significant. *The American Statistician*. 2006;60:328–31. doi: 10.1198/000313006X152649

2 Wasserstein RL, Lazar NA. The ASA Statement on p-Values: Context, Process, and Purpose. *The American Statistician*. 2016;70:129–33. doi: 10.1080/00031305.2016.1154108

3 ICMJE | Recommendations | Preparing a Manuscript for Submission to a Medical Journal. https://www.icmje.org/recommendations/browse/manuscript-preparation/preparing-for-submission.html (accessed 15 March 2026)

4 Ioannidis JPA. What Have We (Not) Learnt from Millions of Scientific Papers with P Values? *The American Statistician*. 2019;73:20–5. doi: 10.1080/00031305.2018.1447512

5 Efron B. Bayesians, Frequentists, and Scientists. *Journal of the American Statistical Association*. 2005;100:1–5.

6 Armitage P. Fisher, Bradford Hill, and randomization. *Int J Epidemiol*. 2003;32:925–8; discussion 945-948. doi: 10.1093/ije/dyg286

7 Fisher RA. Uncertain Inference. *Proceedings of the American Academy of Arts and Sciences*. 1936;71:245–58. doi: 10.2307/20023225

8 Fisher RA. *The design of experiments*. Oxford, England: Oliver & Boyd 1935.

9 Kubiak AP, Kawalec P. Prior Information in Frequentist Research Designs: The Case of Neyman's Sampling Theory. *J Gen Philos Sci*. 2022;53:381–402. doi: 10.1007/s10838-022-09600-x

10 Greenland S. Tests for interaction in epidemiologic studies: a review and a study of power. *Stat Med*. 1983;2:243–51. doi: 10.1002/sim.4780020219

11 Statistical Rituals: The Replication Delusion and How We Got There - Gerd Gigerenzer, 2018. https://journals.sagepub.com/doi/10.1177/2515245918771329 (accessed 15 March 2026)

12 Gigerenzer G, Marewski JN. Surrogate science: The idol of a universal method for scientific inference. *Journal of Management*. 2015;41:421–40. doi: 10.1177/0149206314547522

13 Baduashvili A, Evans AT, Cutler T. How to understand and teach P values: a diagnostic test framework. *J Clin Epidemiol*. 2020;122:49–55. doi: 10.1016/j.jclinepi.2020.03.003

14 Browner WS, Newman TB. Are all significant P values created equal? The analogy between diagnostic tests and clinical research. *JAMA*. 1987;257:2459–63.

15 Agarwal A, Santesso N, Agoritsas T, *et al.* Crossing the null does not mean "no effect": a survey of internal medicine physicians on the interpretation of effect estimates with wide confidence intervals. *Journal of Clinical Epidemiology*. 2026;194:112206. doi: 10.1016/j.jclinepi.2026.112206

16 Windish DM, Huot SJ, Green ML. Medicine residents' understanding of the biostatistics and results in the medical literature. *JAMA*. 2007;298:1010–22. doi: 10.1001/jama.298.9.1010

17 Hauer E. The harm done by tests of significance. *Accid Anal Prev*. 2004;36:495–500. doi: 10.1016/S0001-4575(03)00036-8

18 Rafi Z, Greenland S. Semantic and cognitive tools to aid statistical science: replace confidence and significance by compatibility and surprise. *BMC Med Res Methodol*. 2020;20:244. doi: 10.1186/s12874-020-01105-9

19 Hill AB. The environment and disease: association or causation? *Proc R Soc Med*. 1965;58:295–300. doi: 10.1177/003591576505800503

20 Gigerenzer G. Statistical Rituals: The Replication Delusion and How We Got There. *Advances in Methods and Practices in Psychological Science*. 2018;1:198–218. doi: 10.1177/2515245918771329

21 ICH E9 statistical principles for clinical trials - Scientific guideline | European Medicines Agency (EMA). 1998. https://www.ema.europa.eu/en/ich-e9-statistical-principles-clinical-trials-scientific-guideline (accessed 15 March 2026)

22 Temple R. How FDA currently makes decisions on clinical studies. *Clinical Trials*. 2005;2:276–81. doi: 10.1191/1740774505cn097oa

23 Neyman, Jerzy. Silver jubilee of my dispute with Fisher. *Journal of the Operations Research Society of Japan*. 1961;3:1961.

24 Hurlbert SH, Lombardi CM. Final Collapse of the Neyman-Pearson Decision Theoretic Framework and Rise of the neoFisherian. *Annales Zoologici Fennici*. 2009;46:311–49. doi: 10.5735/086.046.0501

25 Cobb G. Mere Renovation is Too Little Too Late: We Need to Rethink our Undergraduate Curriculum from the Ground Up. *The American Statistician*. 2015;69:266–82.

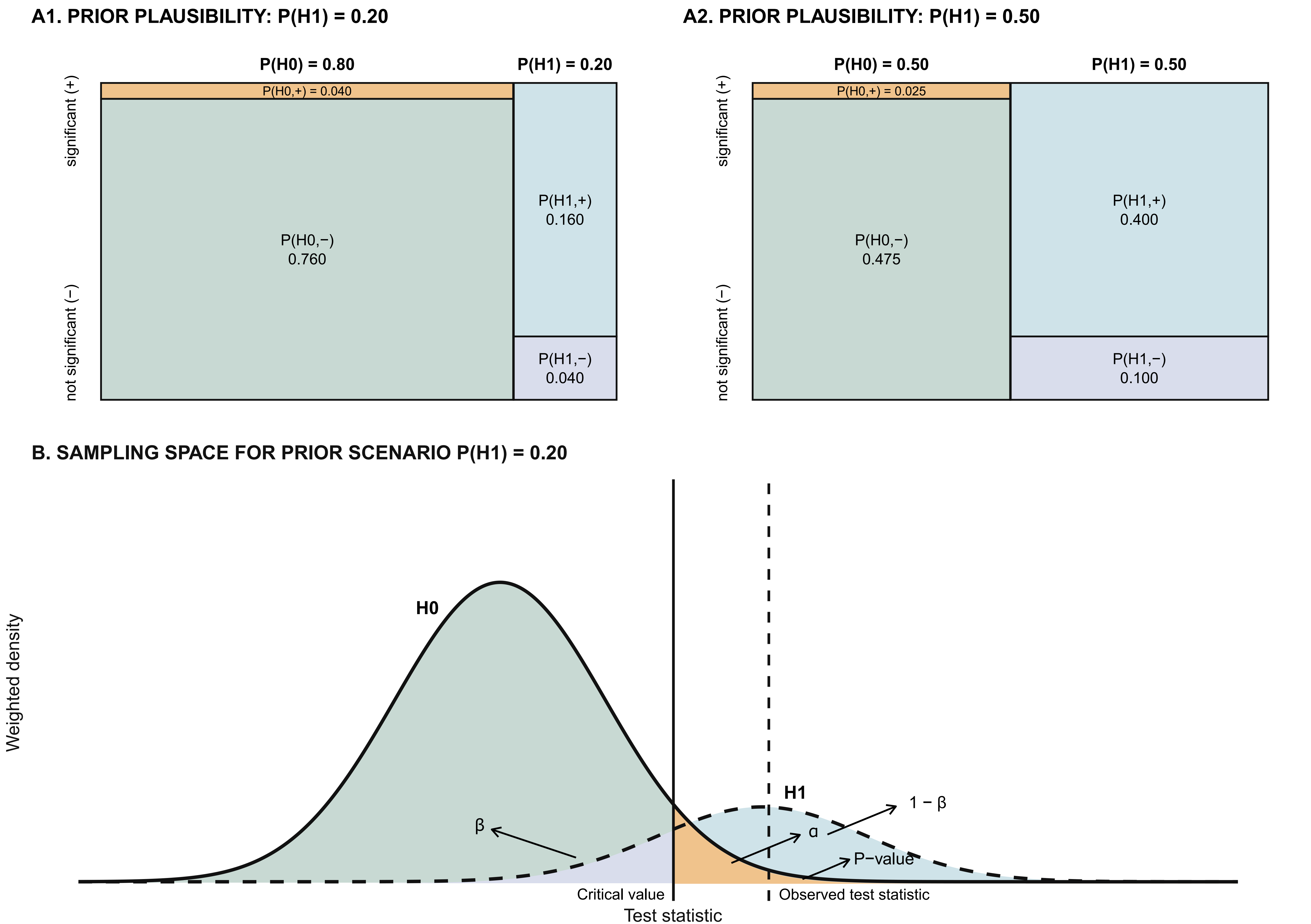
A1. PRIOR PLAUSIBILITY: P(H1) = 0.20
P(H0) = 0.80
P(H1) = 0.20
significant (+)
not significant (−)
P(H0,+) = 0.040
P(H0,−)
0.760
P(H1,+)
0.160
P(H1,−)
0.040
A2. PRIOR PLAUSIBILITY: P(H1) = 0.50
P(H0) = 0.50
P(H1) = 0.50
significant (+)
not significant (−)
P(H0,+) = 0.025
P(H0,−)
0.475
P(H1,+)
0.400
P(H1,−)
0.100
B. SAMPLING SPACE FOR PRIOR SCENARIO P(H1) = 0.20
Weighted density
H0
H1
β
α
1 − β
P−value
Critical value
Observed test statistic
Test statistic

A

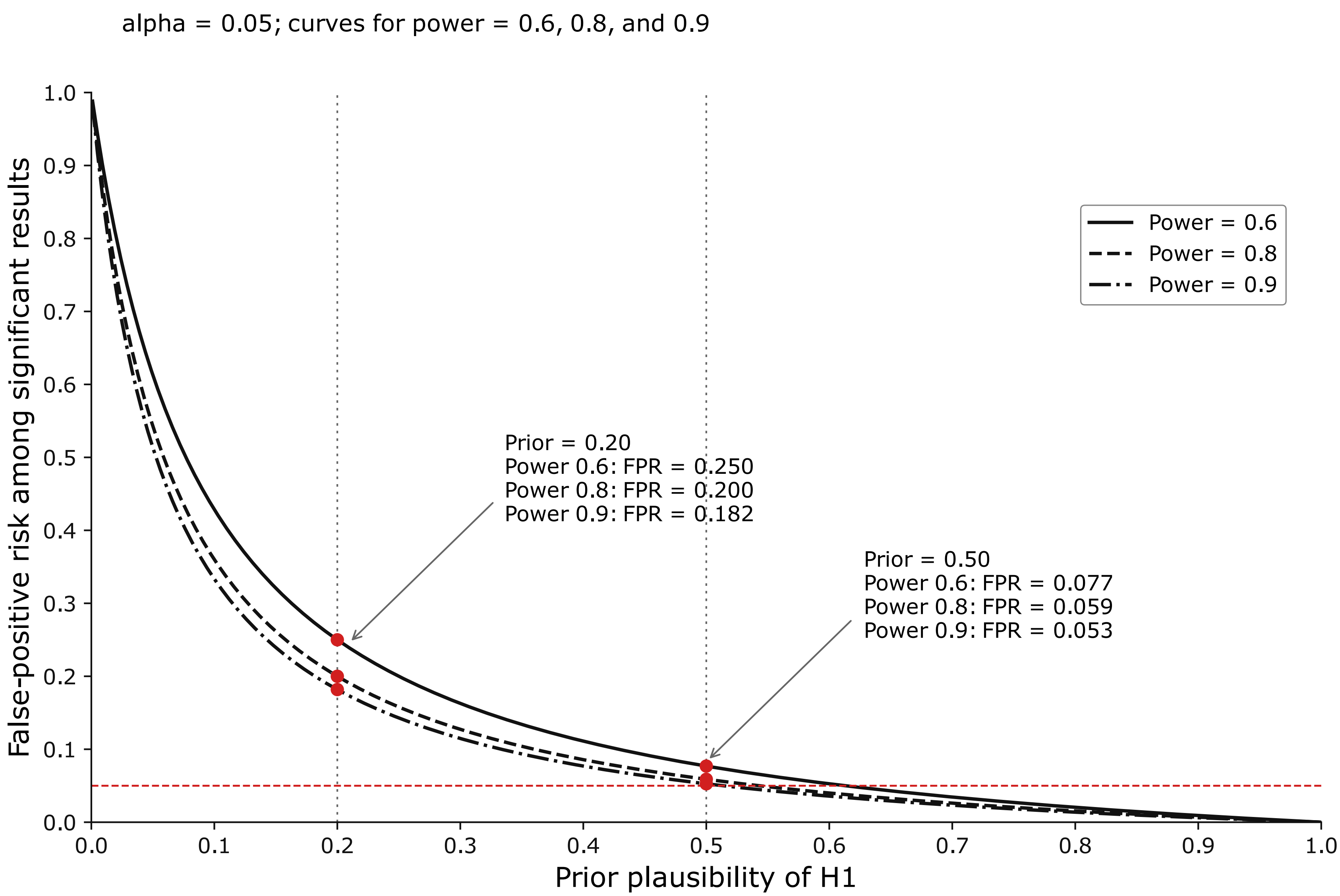

B

Logical function	Diagnostic test	RCT	Link to figure
Prior probability	Prevalence P(D+)	Plausibility P(H1)	x-axis (0 to 1)
Power / detection	Sensitivity P(T+|D+)	Power 1−β	Three curves (0.6, 0.8, 0.9)
Type I error	1−Specificity P(T+|D-)	α	Fixed: α = 0.05
Posterior (positive result)	PPV P(D+|T+)	1−FPR	y-axis; red annotated points

C

Parameter	Diagnostic test	RCT	Figure read-out
Prior	Prev. = 0.20	P(H1) = 0.20	x = 0.20 (dotted line)
Power	Se = 0.80	1−β = 0.80	Dashed curve
Type I error	1−Sp = 0.05	α = 0.05	Fixed parameter
Posterior	PPV = 0.80	1−FPR = 0.80	Red point: FPR = 0.200
